# Acoustically Detonated Microbubbles Coupled with Low Frequency Insonation: Multiparameter Evaluation of Low Energy Mechanical Ablation

**DOI:** 10.1021/acs.bioconjchem.1c00203

**Published:** 2021-07-19

**Authors:** Mike Bismuth, Sharon Katz, Hagar Rosenblatt, Maayan Twito, Ramona Aronovich, Tali Ilovitsh

**Affiliations:** † Department of Biomedical Engineering, Tel Aviv University, Tel Aviv 6997801, Israel; ‡ The Sagol School of Neuroscience, Tel Aviv University, Tel Aviv 6997801, Israel

## Abstract

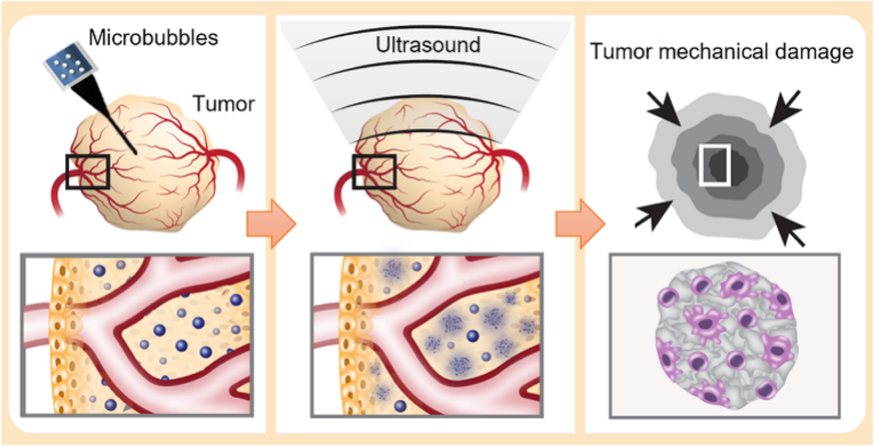

Noninvasive ultrasound surgery can be achieved using focused ultrasound to locally affect the targeted site without damaging intervening tissues. Mechanical ablation and histotripsy use short and intense acoustic pulses to destroy the tissue via a purely mechanical effect. Here, we show that coupled with low-frequency excitation, targeted microbubbles can serve as mechanical therapeutic warheads that trigger potent mechanical effects in tumors using focused ultrasound. Upon low frequency excitation (250 kHz and below), high amplitude microbubble oscillations occur at substantially lower pressures as compared to higher MHz ultrasonic frequencies. For example, inertial cavitation was initiated at a pressure of 75 kPa for a center frequency of 80 kHz. Low frequency insonation of targeted microbubbles was then used to achieve low energy tumor cell fractionation at pressures below a mechanical index of 1.9, and in accordance with the Food and Drug Administration guidelines. We demonstrate these capabilities in vitro and in vivo. In cell cultures, cell viability was reduced to 16% at a peak negative pressure of 800 kPa at the 250 kHz frequency (mechanical index of 1.6) and to 10% at a peak negative pressure of 250 kPa at a frequency of 80 kHz (mechanical index of 0.9). Following an intratumoral injection of targeted microbubbles into tumor-bearing mice, and coupled with low frequency ultrasound application, significant tumor debulking and cancer cell death was observed. Our findings suggest that reducing the center frequency enhances microbubble-mediated mechanical ablation; thus, this technology provides a unique theranostic platform for safe low energy tumor fractionation, while reducing off-target effects.

## Introduction

The National Cancer Institute estimates that 1.8 million new cases of cancer will be diagnosed in the USA in 2020 and over 600,000 patients will die from the disease.^1^ Breast cancer is the most common solid tumor in women, accounting for more than 25% of all cancer-related deaths.^1^ Surgical resection is the most frequently selected intervention, because minimization of cancerous tissues renders immunotherapies and chemotherapies more effective.^2^ Nevertheless, surgery is an invasive procedure that carries a risk for the patient; thus, alternative noninvasive surgical techniques are greatly needed. Among these techniques, focused ultrasound (FUS) is a versatile, noninvasive, clinically adopted therapy method.^3^ Compared to other ablation techniques such as radiofrequency ablation,^4^ microwave ablation,^5^ laser ablation,^6^ and cryosurgery,^7^ ultrasound (US) is noninvasive and cost-effective and offers a high penetration depth.^8^ Conversely, low frequency FUS (below 650 kHz) has gained a lot of interest in recent years, as it is capable of penetrating through an intact human skull with reduced attenuation and distortion, while focusing the ultrasonic energy deep into the brain,^9,10^ opening the door to noninvasive brain therapy.^11^

**Figure 1:**
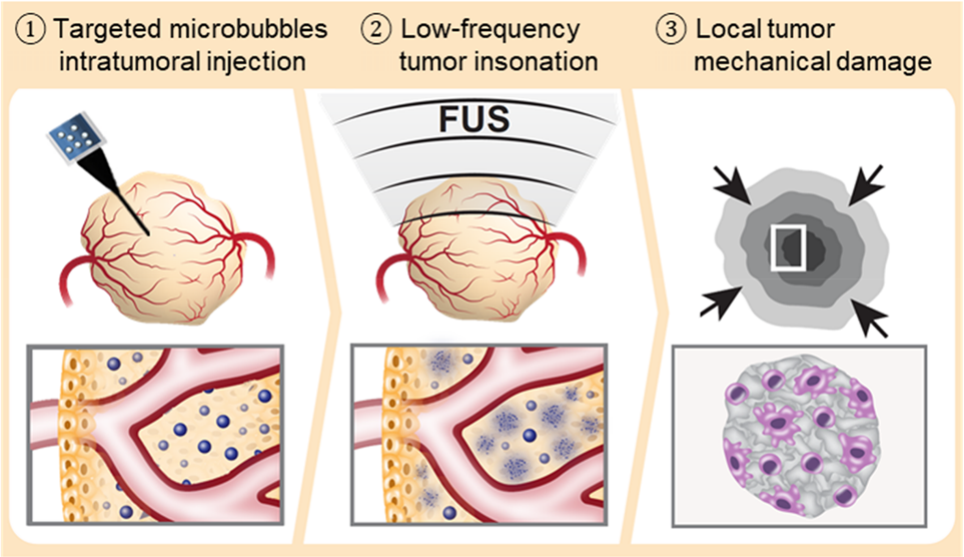
Schematic illustration of the proposed method. Tumor-targeted microbubbles are intratumorally injected into a tumor, followed by the application of low frequency focused ultrasound to detonate the microbubbles, reducing tumor cell viability and performing low energy mechanical ablation.

Noninvasive US surgery can be conducted via two main mechanisms.^12^ The first is thermal ablation where the US beam is focused to a small region of interest, leading to a temperature increase and causing cell death through heat.^13^ However, these treatments are prolonged and costly, because they require magnetic resonance thermometry. More importantly, precise and predictable thermal treatment of deep-seated tissues without affecting complex intervening tissue layers and healthy surrounding tissues is challenging.^14^ Alternatively, histotripsy is a local noninvasive and nonthermal US surgery method that uses high-intensity FUS energy (tens of MPa in pressure) to mechanically ablate deep tissues, fractioning the targeted soft tissue into subcellular debris in the form of liquid using very short, focused, high-pressure US pulses, while leaving the surrounding organs and tissues unaffected.^15^ While histotripsy was shown to clinically treat both benign and malignant conditions,^16^ conventional histotripsy raises safety concerns because of the need to focus such a high energy into the body, as well as the potential for off-target effects.^10^ For example, leg muscle damage and edema resulting from histotripsy ablation of hepatocellular carcinoma have been reported in an in vivo study near the treated region.^17^ Respiration-motion can lead to incomplete ablation or collateral damage and considerably alter precision and efficacy.^16^ Further, the need to fabricate high intensity focused transducers and the technological challenges associated with it are yet another limitation.^18^ In an effort to reduce the pressure threshold required for histotripsy, the combination of histotripsy with microbubbles (MB) or nanodroplets was proposed; however, in the megahertz US range, the combination resulted in a 2- to 3-fold reduction in the onset pressure to ∼10 MPa, which is still a high pressure.^19−23^ The combination was also proposed in the context of brain therapy and the creation of spine injury models.^24,25^ In this paper, we developed a therapeutic platform for low-energy, minimally invasive, US surgery of tumors using MBs, with an order of magnitude reduction in the required pressure compared to standard histotripsy (Figure 1).

MBs, composed of a gas core and a stabilizing shell, are used as theranostic US agents.^19,26^ Upon US excitation, MBs oscillate, facilitating therapeutic applications such as sonoporation-mediated drug delivery, gene-based therapy,^27,28^ and blood brain barrier opening.^29^ MBs are typically excited by US frequencies that are used for imaging (2–10 MHz).^30^ However, it was recently shown that when the MBs are excited by a frequency of 250 kHz (an order of magnitude below the resonance frequency of these agents); their oscillations are significantly enhanced, facilitating low energy blood brain barrier opening and gene delivery to tumors.^31,32^ The Blake threshold effect is the physical phenomenon that triggers the large expansion of MBs well below their resonance frequency.^33−35^ Aside from the enhanced MB vibrational response, the use of low transmission frequency enhances the penetration depth because of the reduced tissue absorbance at this frequency range, which minimizes attenuation compared to higher frequencies.^9,31,36^ Further, the low frequency enlarges the focal zone which aids in treating larger volumes simultaneously. Here, we sought to utilize the high amplitude MB oscillations and use them as cavitation nuclei for low energy histotripsy of breast cancer tumors in vivo while operating below a mechanical index (MI) of 1.9 in accordance with the Food and Drug Administration (FDA) guidelines.

The paper is organized as follows. First, we used theoretical predictions based on the Marmottant model^37^ to compare MB expansion ratio as a function of the US center frequency excitation (2000, 250, and 80 kHz). Next, a multiparameter evaluation was carried using a dual imaging-therapy setup to evaluate MB cavitation in tissue-mimicking phantoms both for free MBs and for cell-targeted MBs (TMB), providing experimental validation to the numerical simulations. The impact of TMB oscillations on cell viability was optimized in a suspension of cultured cancer cells to demonstrate cell fractionation in vitro. Finally, in vivo TMB-mediated mechanical ablation was performed in a murine breast cancer model in mice.

## Results

### Marmottant Model Simulation Results

**Figure 2:**
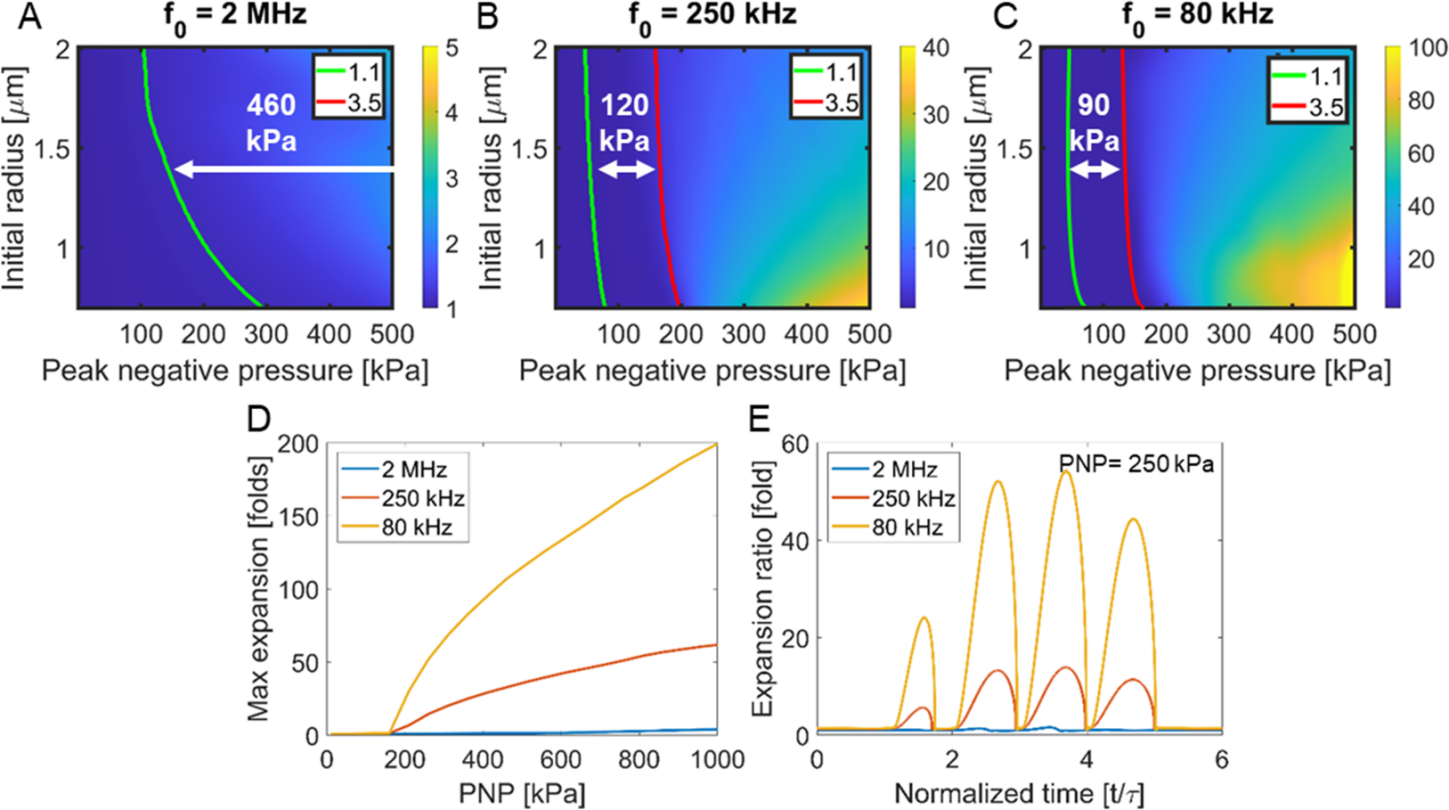
Theoretical prediction of microbubble expansion ratio. Expansion ratio as a function of the peak negative pressure (PNP) and microbubble (MB) initial radius for a center frequency of (A) 2 MHz, (B) 250 kHz, and (C) 80 kHz. The green and red lines indicate an expansion ratio of 1.1- and 3.5-fold, respectively. (D) Maximal expansion ratio as a function of the PNP for the three different center frequencies and for a MB’s initial radius of 0.75 μm. (E) Temporal MB response resulting from a 4-cycle excitation at a PNP of 250 kPa for the three frequencies and an initial MB radius of 0.75 μm.

MB expansion ratio was predicted through numerical simulations, for peak negative pressures (PNP) ranging from 0 to 500 kPa, and for center frequencies of 2 MHz, 250 kHz, and 80 kHz. MBs radius varied from 0.75 to 2 μm (to reflect the sizes of commercially available MBs such as SonoVue and Definity) (Figures 2A–C). The stable cavitation range that is associated with expansion ratios between 1.1 and 3.5 is indicated by the red and green lines, respectively. The highest expansion ratio is predicted for the 80 kHz, reaching a factor of 120 at a PNP of 500 kPa, compared to 38 for 250 kHz and 1.4 for 2 MHz. The stable cavitation range is narrowest for the 80 kHz center frequency (90 kPa), compared to 250 kHz (120 kPa) and 2 MHz (460 kPa). Since the MBs used in this paper are 0.75 μm in radius, the predicted maximal expansion ratio as a function of the PNP (0 to 1000 kPa) and the center frequency excitation (2 MHz, 250 kHz, and 80 kHz) for this MB diameter are presented in Figure 2D. For a constant PNP of 250 kPa (that will be later used in the in vivo studies), Figure 2E compares the expansion ratio as a function of time following 4-cycle excitation for the three different center frequencies.

### Tissue Mimicking Phantom Results

**Figure 3:**
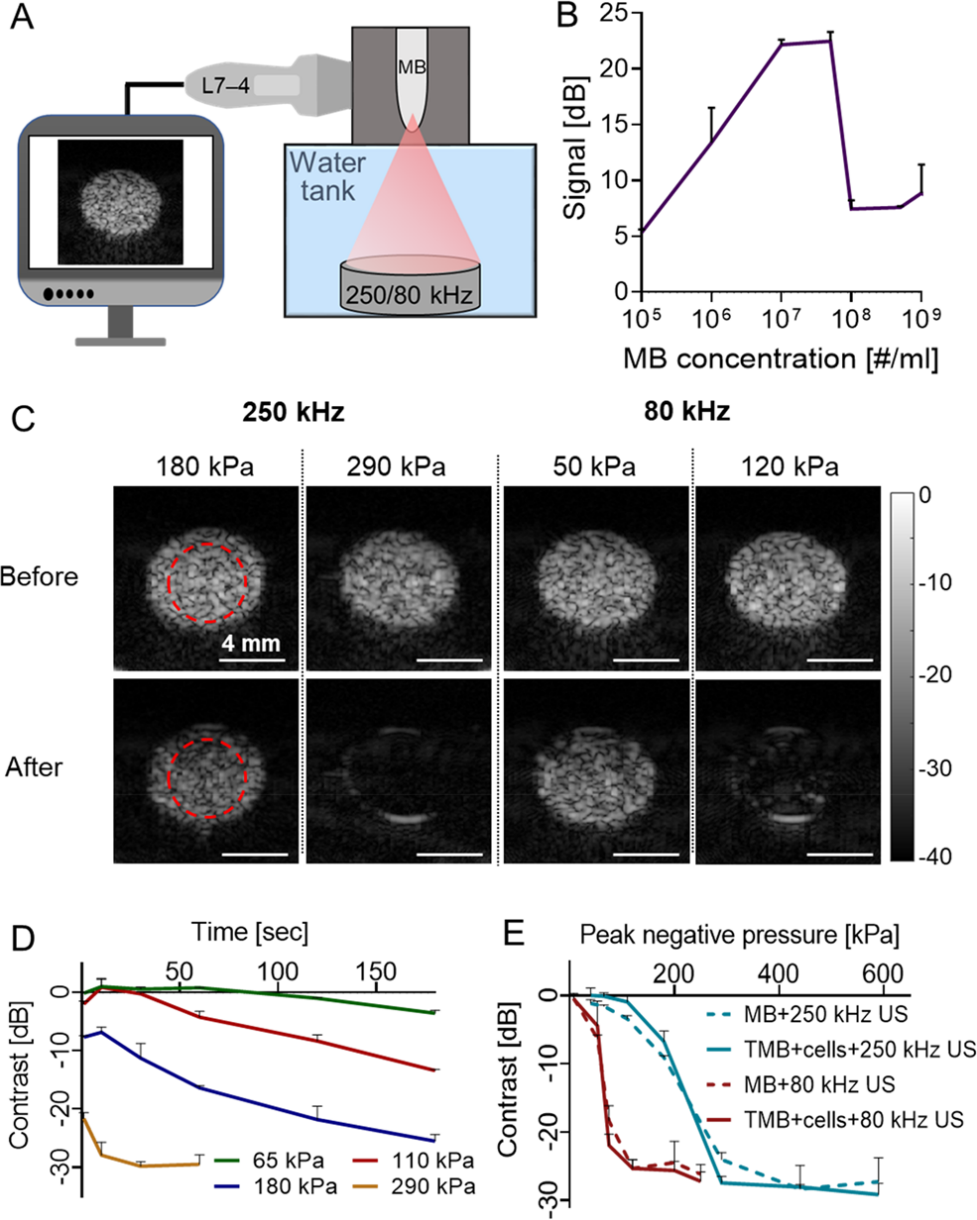
Optimization results in tissue mimicking phantoms. (A) Diluted microbubble (MB) solution is injected into a rod inclusion in an agarose cube that is placed at the focal region of a dual imaging-therapy setup. (B) Inclusion signal as a function of MB concentration. (C) Ultrasound (US) images of the MB-filled inclusion before and after application of a therapeutic US treatment with center frequencies of 250 kHz (either 180 or 290 kPa) and 80 kHz (either 50 or 120 kPa). (D) Impact of treatment duration on the contrast reduction for peak negative pressures (PNP) of 65, 110, and 180, representing stable cavitation, and 290 kPa, representing inertial cavitation and a center frequency of 250 kHz. (E) Contrast reduction as a function of PNP for MB only and cells + targeted MBs (TMB), for the two center frequencies. All experiments were performed in triplicate. All data are plotted as mean ± SD.

The aim of the tissue mimicking phantom experiments was to affirm the numerical simulations via an experimental observation. The experiments include the application of low-frequency insonation to a MB-filled inclusion, while evaluating the impact of insonation parameters on the inclusion contrast using a dual imaging-therapy setup (illustrated in Figure 3A). The imaging transducer was used to capture the inclusion image before and after therapeutic US application. When MB oscillate in inertial cavitation, they are fragmented, and as a result, their contrast is reduced. Thus, analyzing the inclusion contrast as a function of the US parameters is an indicator of the MBs status. Initially, optimal MB concentration was selected by evaluating the signal of the MB suspension as a function of the MB concentration. A value of 1 × 10^7^ MBs/mL yielded an optimal signal and hence was used in the following experiments (Figure 3B). Lower concentrations yield reduced signal due to a lower echogenicity of the MB solution, while at higher concentrations, US signal was blocked by the MBs, reducing the overall contrast (Figure 3B).

Next, in order to assess the effect of the low frequency US excitation on MB contrast reduction, US imaging was used to acquire an image before and after a 1 s US treatment (Figure 3C). Prior to low frequency US application, contrast was maximal (0 dB). Then, low frequency US was applied to the inclusion with different PNPs, treatment durations, frequencies (80 and 250 kHz), pulse repetition frequencies (PRF), number of cycles, and duty cycles. High contrast reduction is associated with inertial cavitation and MB destruction, which are the parameters required for the mechanical ablation. For example, contrast reduction by over 20 dB is observed for a PNP of 290 kPa @ 250 kHz and 120 kPa @ 80 kHz. In comparison, for a 1 s treatment of 180 kPa @ 250 kHz and 50 kPa @ 80 kHz, the contrast was reduced by 9.1 and 5.8 dB, respectively (Figure 3C). The numerical simulations indicate that inertial cavitation initiates at a PNP of ∼190 kPa for a MB with a 0.75 μm radius for a center frequency of 250 kHz. The aim of the Figure 3D is to assess the impact of contrast reduction for conditions of stable cavitation vs inertial cavitation. For stable cavitation, 3 PNPs were selected: 180 kPa, which is close to the transition to inertial cavitation threshold, and 110 and 65 kPa that are well below the threshold. In addition, 290 kPa was chosen as a PNP well above the inertial cavitation threshold. For the PNP of 290 kPa, a treatment duration of 1 s suffices for significant contrast reduction, thus for Figure 3E, a treatment duration of 1 s was selected. However, for the stable cavitation PNPs, the MB destruction mechanism is not due to fragmentation or collapse, but rather through loss of gas with each oscillation. This is a gradual process that increases as a function of insonation duration, and therefore, the graph slopes of the stable cavitation pressures (65, 110, and 180 kPa) decrease linearly. Since 180 kPa is closest to the inertial cavitation threshold, it yields the maximal signal reduction (21.6 dB) following a 3 min treatment, compared to the maximal signal reduction of 65 kPa (3.6 dB) and 110 kPa (13.4 dB) (Figure 3D).

For a 1 s treatment duration, contrast reduction to a minimal value of −25 dB occurred at a substantially lower PNP at a center frequency of 80 kHz compared to 250 kHz (120 kPa, MI of 0.42 vs 290 kPa, MI of 0.58) (Figure 3E). Subsequently, the same treatment parameters were applied to TMBs bound to 4T1 breast cancer cells in order to assess the impact of MB targeting to cells on the contrast reduction results. Results were similar between free MBs and cells + TMB, indicating a steeper reduction in contrast when using the 80 kHz center frequency, compared to 250 kHz (Figure 3E). Notably, in Figure 3E we matched the amount of the number of cycles (125 cycles) for both the 250 kHz and 80 kHz. However, this results in a longer temporal pulse length for the 80 kHz frequency. Therefore, to match the temporal pulse length and duty cycle of the 80 kHz to that of the 250 kHz, we decreased the number of cycles from 125 cycles to 40 cycles for the 80 kHz. In this case, the pulse length for both 80 kHz and 250 kHz was 0.5 ms, and the duty cycle was 1.5%. Contrast reduction was then assessed as a function of PNP for 80 kHz insonation with 40 cycles compared with 125 cycles, and a PRF of 30 Hz. No significant differences were observed due to the decrease of the number of cycles to 40 (Figure S1). Lastly, the effects of the pulse length, PRF, and thus also the duty cycle were evaluated. For insonation at 80 kHz, 250 kPa, and a PRF of 30 Hz, reducing the number of cycles from 40 cycles (duty cycles of 1.5%) to 20 cycles (duty cycle of 0.75%) did not alter contrast reduction (contrast reduction remained ∼ −26 dB). However, reducing the number of cycles from 40 to 10 cycles (duty cycle of 0.375%) reduced the contrast to ∼ −20 dB (not significant (*p* > 0.05), Figure S2). Comparing PRFs of 30, 20, and 10 Hz, which corresponds to duty cycles of 1.5%, 1%, and 0.5%, respectively, did not affect contrast reduction (Figure S3). Based on the results, we can conclude that using a duty cycle beyond 0.5% yields optimal contrast reduction.

### In Vitro Nonthermal Ablation Results

**Figure 4:**
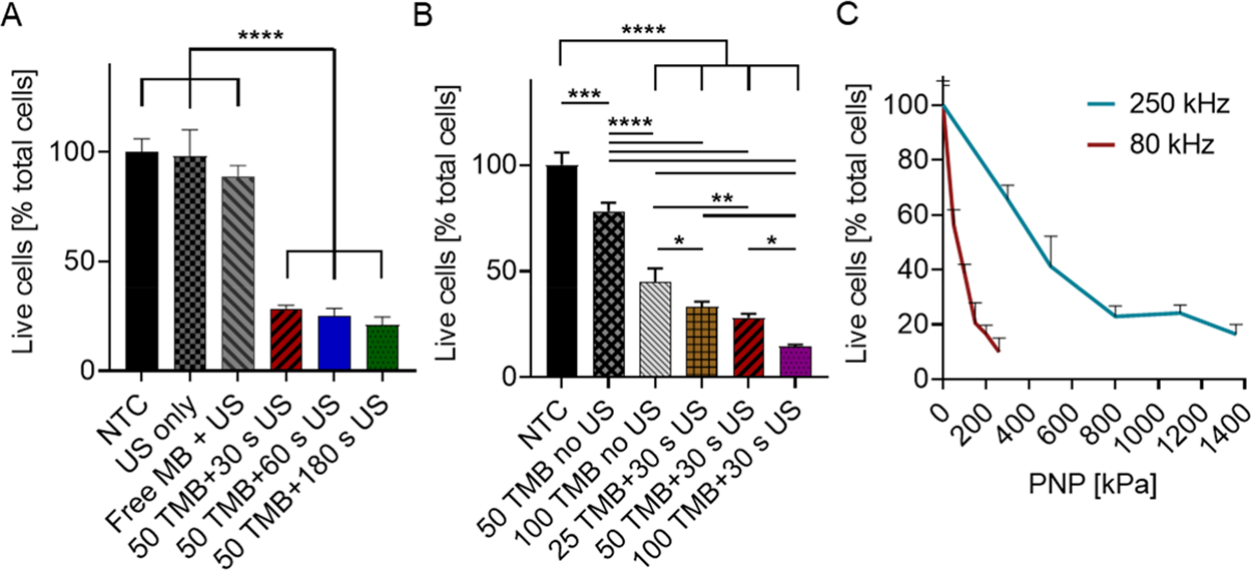
In vitro ultrasound-mediated nonthermal ablation. (A) Treatment duration optimization comparing cell viability following 30, 60, and 180 s ultrasound (US) treatment durations at a peak negative pressure (PNP) of 500 kPa, center frequency of 250 kHz, and 50 targeted microbubbles (TMB) per cell. Control groups include a no treatment group (NTC), US treatment only (US-only), and nontargeted microbubbles with US treatment (Free MB + US). (B) Impact of the number of TMB (25, 50, and 100 TMBs) per cell on cell viability. Treatment includes cells + 30 s US (250 kHz, 500 kPa) + TMB. Control groups include NTC, and TMB only (no US) with 50 or 100 TMB/cell. (A,B) One-way ANOVA with Tukey’s multiple comparisons test. Adjusted *p* values were *p < 0.05, **p < 0.01, ***p < 0.001, and ****p < 0.0001. (C) Cell viability as a function of applied PNP for 80 kHz and 250 kHz US with 50 TMBs/cell and a treatment duration of 30 s. All data are plotted as mean ± SD.

Low frequency US-mediated in vitro experiments assessed the impact of TMB oscillations on cell viability as a function of the PNP and center frequency. Initially, treatment duration and TMB concentration were optimized. For a constant ratio of 50 TMBs per cell, center frequency of 250 kHz, and a PNP of 500 kPa, no significant difference was found between the different treatment durations (30, 60, and 180 s), where all reduced cell viability to ∼24.8% of live cells (Figure 4A). Cell viability remained similar in all of the control group of no treatment control (NTC), US only, and untargeted MBs + 500 kPa US (*p* < 0.0001 compared to treated groups). Since cell viability remained similar for all of the treatment durations tested, the shortest treatment of 30 s was chosen for the following experiments. The next parameter that was optimized was the TMB concentration. Different concentrations (25, 50, and 100 TMBs per cell) were compared. Increasing the TMB concentration per cell reduced viability to a value of 14 ± 0.8% of live cells for the 100 TMB/cell, as compared to 25 TMB/cell that yielded 33.4 ± 2.3% viability (*p* < 0.01) (Figure 4B). However, viability for the control group that contained only 100 TMBs per cell (without US) was also reduced to 44.9 ± 6.5% (*p* < 0.0001 compared to the treated group). In comparison, a concentration of 50 TMB/cell + US treatment yielded a 28.2 ± 1.8% viability, while the control of 50 TMB/cell without US was 78 ± 4% (*p* < 0.0001 compared to the treated group). Due to the enhanced viability in the control group, a concentration of 50 TMBs per cell was selected for the following experiments.

Effective binding rate for a concentration of 50 TMB/cell was evaluated via microscopy, resulting in an active binding rate of 19.4 ± 3 TMBs/cell. Thus, binding efficacy was 38.8 ± 6%, assuming that the dose that was added to each vial was 50 TMB/cell. Lastly, comparing cell viability as a function of the PNP for center frequencies of 250 and 80 kHz shows a rapid reduction in cell viability for the center frequency of 80 kHz compared to 250 kHz (Figure 4C). Five PNPs were tested for each treatment, spanning 300 to 1360 kPa for the center frequency of 250 kHz and 50 to 260 kPa for the center frequency of 80 kHz. An average viability of 22.9 ± 3.8% was obtained with the 250 kHz treatment at 800 kPa (MI = 1.6), while similar viability was achieved for the frequency of 80 kHz at 150 kPa (MI = 0.53).

### In Vivo Ablation Treatment Results

**Figure 5:**
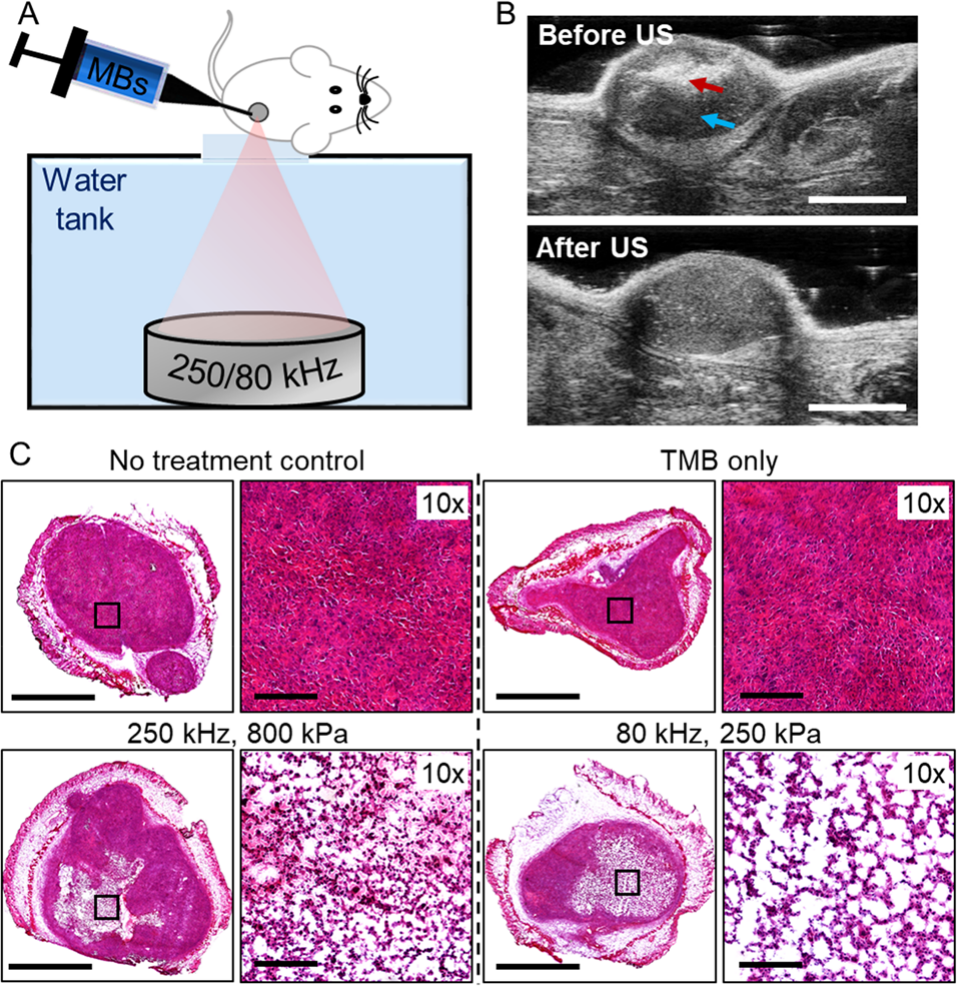
Tumor-targeted microbubbles combined with low frequency ultrasound generates mechanical damage in vivo. (A) Targeted microbubbles (TMB) are intratumorally injected, followed by low frequency ultrasound (US) application in a custom-made setup. (B) Representative US images before and after therapeutic US application. TMBs location and dark shade generated by the TMBs are marked with red and blue arrows, respectively. Scale bar is 4 mm. (C) Histological photomicrographs for no-treatment control (NTC), only TMB (no US), and directly treated US + TMB tumors treated with 250 or 80 kHz US. Scale bars are 2 mm for tumor cross sections and 200 μm for 10× images.

The impact of low frequency TMB oscillations on breast cancer tumors was evaluated in vivo on bilateral breast cancer tumor-bearing mice, to compare the effect of 250 kHz excitation compared to a center frequency of 80 kHz. US was applied to the tumors following an intratumoral (IT) injection of a TMB suspension (Figure 5A). After the IT injection, US imaging confirmed the presence of TMB in the tumors (Figure 5B red arrow). Notably, the TMB blocks the propagation of the US beam, casting a dark shade within the tumor (Figure 5B, blue arrow). US imaging before and after low frequency therapeutic US treatment confirmed complete TMB destruction post insonation (Figure 5B). The PNP values for the 250 kHz (800 kPa, MI = 1.6) and for the 80 kHz (250 kPa, MI = 0.9) were chosen to maintain a constant cavitation index (CI) of 3.2, while operating below the FDA MI upper limit of 1.9.

Histological evaluation performed 24 h post US treatment confirmed the presence of defined lesions with an average diameter of 2.5 mm in the tumors, that were not visible in control groups (Figure 5C). 10× magnified images of the lesion region indicate a larger degree of tissue perforation with the 80 kHz treatment. Quantification of the total white area in the magnified lesion images, corresponding to the tissue generated holes, resulted in an average of 48.6 ± 6.8% of tissue perforation for the 80 kHz, compared to 31.3 ± 3.8% for the 250 kHz (*p* < 0.05).

## Discussion

The development of minimally invasive ablation techniques for local tumor treatment, as an alternative to invasive tumor resection, is a growing field with high clinical applicability.^38,39^ US-based ablation techniques allow less pain, shorter recovery time, and treatment of patients that are ineligible for surgical resection due to the location of the tumor, age, or presence of other medical conditions.^6,40,41^ Due to the fast development of imaging modalities and devices, US tumor ablation has the advantage to be image-guided in many cases.^42,43^ Our focus here is on mechanical tumor fractionation via histotripsy, where our aim is to reduce the required energy for standard histotripsy by over an order of magnitude through the use of TMB coupled with low frequency US.

The proposed method requires a careful selection of protocol components including US parameters (center frequency, PNP, PRF, duty cycle, and treatment duration), TMB formulation, and concentration. Our results confirm that TMB oscillations are enhanced at 80 kHz insonation compared to 250 kHz, despite having a lower MI. Thus, tumor debulking and reduced viability can be achieved at a PNP of 250 kPa for the 80 kHz.

The aim of the tissue mimicking phantom experiments was to affirm the numerical simulations via an experimental observation. The numerical simulations of MB oscillations provide the MBs’ expansion ratio, where a threshold of 3.5 is the estimated inertial cavitation threshold above which the MBs will fragment and collapse. The tissue mimicking phantom experiments characterize MB’s destruction as a function of the PNP, in correlation with the values derived from the numerical simulations. Moreover, in vitro, the MBs were targeted to the cells, which is a different condition compared to free MBs. Therefore, in the tissue mimicking phantom experiment, we also compared the effect of insonation of cell-TMBs when attached to cells, compared to free MBs. Our results show that due to the low frequency insonation, the effect of cell targeting is not significant, and hence the same parameters can be used for the in vitro experiments. This approach facilitates the multiparameter evaluation of insonation parameters and MB concentration.

In vitro, molecular targeting of MBs to breast cancer cells was essential for effective cell detonation. In the in vitro studies, a concentration of 50 TMBs per cell and treatment duration of 30 s were chosen. Increasing treatment duration to 180 s did not affect cell viability, and therefore, a 30 s treatment was chosen to minimize US exposure. Increasing TMB concentration further reduces cell viability; however, cell viability was also reduced in the TMB only control groups. This might be attributed to the phospholipids or the antibody. At high phospholipid concentration, previous studies reported in vitro cytotoxicity.^44^ Moreover, cytotoxicity of the EPCAM targeted antibody was also reported at high concentrations.^45^ Taken together, both can account for the minor cell toxicity of the TMBs in vitro. Nevertheless, cells are much more sensitive in vitro, without the supporting biological environment in vivo. It should be noted that in vivo no cell death or off-target toxicity was observed in the only TMB control.

While the MI is a metric for predicting mechanical bioeffects as a result of cavitation and has an upper limit of 1.9 based on the FDA guidelines,^46^ the CI is an indicator for gauging the level of MB cavitation.^47^ Thus, the impact of US frequency on cell viability was evaluated for a constant CI of 3.2 (800 kPa for 250 kHz and 250 kPa for 80 kHz). Using these parameters, cell viability was reduced to 16% for the center frequency of 250 kHz (MI of 1.6), compared to 10% viability for a PNP of 250 kPa (MI of 0.9).

The optimization and multiparameter evaluation process was performed in the numerical simulations, tissue mimicking phantom experiments, and in vitro experiments as a prerequisite step prior to the in vivo experiments. Following optimization, the optimal parameters were chosen for the in vivo experiments, and resulted in an effective low energy, MBs-based histotripsy of the tumors. In vivo, the combination of IT injected TMB followed by low frequency insonation (CI of 3.2) reduced tumor viability, debulked tumor mass, and created defined lesions with large pores in the treated region, as observed on histology. US imaging was used to image tumor-injected TMB, before and following low frequency US treatment, and confirmed TMB destruction. Quantification of the perforated region on histology shows a 55% increase in pore size for the 80 kHz frequency compared to 250 kHz (*p* < 0.05). These results suggest that despite the fact that a center frequency of 80 kHz has a similar CI and a lower MI compared to 250 kHz, higher mechanical damage, and tumor cell death is obtained with 80 kHz. Thus, efficient low energy TMB-mediated mechanical tissue fractionation is enhanced at lower frequencies. The use of low frequency insonation is significant in order to enhance the penetration depth and enlarge the focal zone, while the use of locally injected TMB reduces the off-target risk that exists in standard histotripsy.

Histotripsy is a well-characterized method, and existing literature contains many examples of histotripsy.^16−18,48^ The high energy used in histotripsy is well above the MI, and therefore the US focus will fractionate any tissue that it will encounter. Patient movement as a result of breathing is a challenge that can cause damage to healthy tissues near the focal spot. Therefore, most of the standard histotripsy procedures are performed with a higher center frequency.^12^ As a result, the focal spot size is reduced, and mechanical stirring is required in order to cover the treated area. Here, since the TMBs are injected locally and the PNPs used are below the MI, there is a reduced risk for damaging surrounding healthy tissue. Hence, a large focal spot is used, which facilitates patient alignment and shortens treatment duration. Conversely, IT based therapies are commonly used in clinical studies^49,50^ and are beneficial in reducing systemic exposure to the therapeutic agent, by reducing off-target toxicity. Reported studies affirm IT injection usage for target sites accessible to biopsy, strengthening the method’s potential clinical translation.^51^ In the vicinity of IT injection, we previously compared TMBs and free MBs under the condition of IT injection, in research focused on transfection via sonoporation.^32^ The results showed a reduced effect with free-MB compared to TMB; thus, the proximity to the tumor cells using TMB plays a significant role both in vitro and in vivo.

Breast cancer was chosen in this work as it is superficial, which facilitates US alignment, treatment, and monitoring, and thus is a practical model for optimizing the method. It is likely that the method can be adapted to other tumor types as well. Further, the ability to deliver large molecules with sonoporation-mediated treatments is closely linked to the US parameters that are used. The high amplitude oscillations obtained at a center frequency of 80 kHz can therefore be also applied to the field of nonviral gene delivery.^32^ Two different breast cancer cell lines were evaluated in this study in order to emphasize the robustness of the method and its ability to effectively treat multiple breast cancer cell lines. Moreover, the 4T1 cell line used in the in vitro section produces highly metastatic tumors that can metastasize to the lung, liver, lymph nodes, and brain.^52^ In this study, we wanted to assess the impact of the treatment on the primary tumor. Thus, for the in vivo studies we chose to focus on a cell line that does not produce metastases quickly (Met-1). In our future studies, we will investigate how the method affects the immune response and whether it can be combined with adjuvant treatments to treat metastatic breast cancer.^53^ For these studies, 4T1 cells will be used for the in vivo model.

While this study optimized TMB-mediated mechanical damage in tumors, standard cancer histotripsy treatments have recently shown promising abilities to stimulate the immune system by releasing tumor-associated antigens, enhancing dendritic cell infiltration to tumors, increasing CD8^+^ T-cell responses, and suppressing the formation of distant metastases.^12,32,48,54,55^ A comprehensive study of the method’s effect on innate and adaptive immune system, mechanism, and survival rate is planned.

## Conclusions

High amplitude oscillations of TMB coupled with low frequency excitation at a center frequency of 80 kHz was developed here as a platform for low energy histotripsy of breast cancer tumors. The mechanical effect is triggered by acoustically detonating locally injected TMB attached to cancer cells, yielding tumor fractionation and reducing cell viability, while operating at a MI of 0.9 and a PNP of 250 kPa. Successful low energy TMB-mediated mechanical ablation developed here includes theoretical prediction of MB oscillations, synthesis and concentration optimization of TMBs, optimization of insonation parameters in tissue mimicking phantoms and in cell cultures, and experimental confirmation in vivo. Our findings suggest that reducing the center frequency further enhances MBs oscillations, amplifying MBs-mediated mechanical treatments.

## Experimental Procedures

### MB Oscillations and Cavitation Monitoring

MB oscillations depend on the US parameters. At a low acoustic pressure, MBs are compressed and expanded repeatedly in a process termed stable cavitation.^56^ At higher acoustic pressure MB undergoes inertial cavitation; the MBs disintegrate and fragment into smaller parts or diminish via gas diffusion. Inertial cavitation produces a high level of energy, inducing liquid jets than can lead to acute mechanical damage to the surrounding environment.^57,58^ In accordance with our previous research,^31,32^ the stable cavitation range was defined beyond a MB expansion ratio of 1.1. The crossover between stable and inertial cavitation was defined beyond an expansion ratio of 3.5 (previous predictions ranged from 2.3 to 3.5).^37,59^

The MI, defined as the PNP divided by the square root of the center frequency,^60^ is a parameter used for clinical safety assessment of US. MI indicates the likelihood of adverse mechanical bioeffects (streaming and cavitation), by gauging the PNP for a given US frequency. For diagnostic imaging, it is FDA limited to a value below 1.9. Beyond this value, mechanical damage is expected due to cavitation.^61^ The CI, defined as the PNP divided by the center frequency,^62^ serves as an indicator of MB stable cavitation. This parameter was shown to serve as a valid indicator of the level of FUS-induced blood brain barrier opening.^47,56^ A CI above 0.02 indicates increased risk that the MB oscillate in inertial cavitation.^56^

### Numerical Modeling

The Marmottant model was used to estimate MB oscillations and expansion ratio.^37^ This model is widely used for MB modeling and has good agreement with experimental observations.^31,63^ We have recently shown that it can accurately predict MB oscillations following low frequency excitation of 250 kHz.^31^ This model takes into consideration parameters related to the MB composition, its surrounding medium viscosity and density and excitation wave. All simulations were performed in MATLAB (Mathworks, Natick, MA). The effect of center frequency, PNP, and MB initial radius on oscillation behavior were evaluated. Initial MB radii values ranged from 0.75 to 2 μm. The expansion ratio for each MB initial radius was calculated as a function of varied PNP values between 0 and 500 kPa. Simulations were performed for 3 center frequencies: 2 MHz, 250 kHz, and 80 kHz. The parameters were identical to those in ref (31). The surface tension of the MB outer radius was set to 0.073 N/m (saline) and to 0.04 N/m for the inner radius. Shell density was 1000 kg/m^3^, shell shear modulus was 122 MPa, shell viscosity was 2.5 Pa·s, the shell surface dilatational viscosity was 7.2 × 10^9^ N, and the elastic compression modulus was 0.55 N/m. Shell thickness was set to 1.5 nm.

### Microbubble Preparation

All MBs used in this paper were composed of a phospholipid shell and a perfluorobutane (C_4_F_10_) gas core. Free untargeted MBs were prepared as reported previously.^31,64^ Briefly, the lipids (2.5 mg per 1 mL) disteroylphosphatidylcholine (DSPC) and 1,2-distearoyl-*sn*-glycero-3-phosphoethanolamine-*N*-[methoxy(polyethylene glycol)-2000] (ammonium salt) (DSPE-PEG2K) (Sigma-Aldrich) were combined at a molar ratio of 90:10 and made using a thin film hydration method. A buffer (mixture of glycerol (10%), propylene glycol (10%), and saline (80%) (pH 7.4)) was added to the lipids and sonicated at 62 °C. The MB precursor solution was aliquoted into vials with liquid volume of 1 mL and saturated with perfluorobutane. Upon use, the vials were shaken for 45 s in a vial shaker and purified via centrifugation to remove MBs smaller than 0.5 μm in radii. TMBs were prepared similarly to the method in ref (32). The lipids (2.5 mg per 1 mL) disteroylphosphatidylcholine (DSPC), 1,2-distearoyl-*sn*-glycero-3-phosphoethanolamine-*N*-[methoxy(polyethylene glycol)-2000] (ammonium salt) (DSPE-PEG2K) (Sigma-Aldrich), and 1,2-distearoylsnglycero-3-phosphoethanolamine-*N*-[biotinyl(polyethylene glycol)2000] (DSPE-PEG2000-Biotin) were combined at a molar ratio of 90:5:5 and prepared similarly to the untargeted MBs. Following activation via the vial shaker and purification, 400 μg of streptavidin (Sigma-Aldrich, catalog number: S4762) was added to the MB cake and incubated for 25 min at room temperature on a rotator. Next, the streptavidin modified MBs were purified to remove excess streptavidin. Subsequently, 15 μg of biotinylated anti-mouse CD326 (EpCAM, BioLegend #118203) antibody was added to the streptavidin–MB cake followed by incubation on a rotator and purification as described in the preceding step. The size and concentration of the purified MBs and TMBs were measured with a particle counter system (AccuSizer FX-Nano, Particle Sizing Systems, Entegris, MA, USA). The bubbles were used within 3 h of their preparation. The size distribution and concentration varied by less than 10% between the measurements.

### Ultrasound Setup

The experimental setup (illustrated in Figure 3A) was composed of a 64-mm-diameter spherically focused single-element transducer (H117, Sonic Concepts, Bothell, WA, USA) that was placed at the bottom of a degassed water tank facing upward and focused to a distance of 45 mm. The fundamental frequency of the H117 transducer used in this work is 250 kHz. However, this is a custom transducer that can operate also at an 80 kHz center frequency using a custom-made matching network purchased from Sonic Concept. When working with the 80 kHz matching network, the bandwidth is between 70 kHz and 105 kHz. At 80 kHz, a third of the maximal PNP is obtained compared to the maximal pressure when working at the center frequency of the transducer (250 kHz). The beam pattern measurements using a calibrated hydrophone (NH0500, Precision Acoustics, UK) show a focal width of 18.89 mm and focal length of 92.66 mm for the 80 kHz center frequency configuration. In each experiment, the desired target was placed at the focal spot. For the in vitro assays, it was either an agarose phantom containing the MBs suspension or a 0.5 mL Eppendorf tube with breast cancer cells. In vivo, the tumor was positioned at this focal spot. The transducer pressure was calibrated with the NH0500 wideband needle hydrophone. A transducer power output unit combining an arbitrary waveform generator together with a radiofrequency amplifier (TPO-200, Sonic Concepts, Bothell, WA, USA) was used to generate the desired signal consisting of a sinusoid at a center frequency of 250 kHz or 80 kHz.

### Optimization Experiments in Agarose Phantoms

Tissue mimicking phantom preparation: Agarose powder (A10752, Alfa Aesar, MA, USA) was mixed with deionized water to a 1.5% solution at ambient temperature and heated until all powder was completely dissolved. The solution was then poured into a mold and cooled at ambient temperature. The mold was 3D printed and contained a 6 mm rod inclusion. The phantom was placed at the focal spot of the US setup. In each experiment, a mixture of MBs or TMBs bound to cells were diluted in degassed phosphate buffered saline (PBS) and injected into the rod inclusion. An imaging transducer (L7–4, Philips ATL) controlled by a programmable US system (Verasonics, Vantage 256, Verasonics Inc., Redmond, WA, USA) was used to image the tissue mimicking phantom before and after the application of the low frequency therapeutic US. The imaging transducer was placed perpendicularly to the spherically focused therapeutic transducer (Figure 3A). The red circles in Figure 3C mark the locations that were used in the contrast calculations. The contrast was defined as the difference in brightness before and after therapeutic US treatment at the region of interest (eq 1):
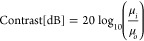
1where μ_*i*_ is the mean of the red circle area after US insonation, and μ_*o*_ is the mean of the same region before US treatment.

### Computation and US Imaging

All of the theoretical predictions and US image analysis were implemented in MATLAB (version 2016b, MathWorks, Natick, MA, USA). The program run on a Dell OptiPlex 7070 PC with a Windows 10 Enterprise 64-bit operating system, Intel Core i7–9700 processor, 3.00 GHz, 16 GB RAM. US imaging in standard two-way focusing was performed using the Verasonics US system, at a center frequency of 5 MHz and with a linear L7–4 imaging transducer. The transducer has 128 elements, with an element size of 7 mm × 0.283 mm (height × width) and a kerf width of 0.025 mm. The excitation for each transmitted pulse was 1 cycle. For MB inertial cavitation optimization experiments, postprocessing of the captured images was performed with Matlab to calculate the contrast reduction as a function of the PNP. In vivo US images were acquired using the Vevo 2100 imaging system (Visualsonics, Canada) at a center frequency of 40 MHz with a linear MS-550D probe operating at a two-way focusing mode.

### In Vitro US-Mediated Ablation Assay

4T1 cells, highly metastatic triple negative murine breast carcinoma cell line,^52^ purchased from ATCC, was used for the in vitro experiments. Cells were cultured in RPMI 1640 supplemented with 10% v/v fetal bovine serum, 1% v/v penicillin–streptomycin, and 0.292 g/L l-glutamine and grown in T75 tissue culture treated flasks until about 85% confluency on the day of the experiment. The 4T1 cells were then collected via dissociation with TrypLE Express (Gibco Corp,12604–013, Grand Island, NY, USA) and resuspended at a concentration of 1 × 10^6^ cells in 300 μL degassed PBS containing calcium and magnesium (PBS+/+). The TMBs were added to the cell mixture according to the differently tested concentrations and incubated for 20 min at room temperature on a rotator allowing the TMBs to bind to the cells.

Following incubation, the mixture of cells and MBs was aliquoted into 0.5 mL Eppendorf tubes. Finally, degassed PBS+/+ was added to a final volume of 0.48 mL per tube and incubated at room temperature for 30 min prior to the US treatment. Next, each Eppendorf tube was placed at the focal spot of the US setup and treated according to the different US treatment parameters tested. Sonication in all of the in vitro studies consisted of a 125 cycle sinusoid with a 250 kHz or 80 kHz center frequency and a PRF of 30 Hz. Initial experiments were aimed to optimize the treatment duration, for tested durations of 30, 60, and 180 s. These experiments were performed with a constant ratio of 50 TMBs per cell and a center frequency of 250 kHz (PNP of 500 kPa). Binding efficacy for the 50 TMBs per cell ratio was evaluated by imaging of the cells after the 20 min incubation with TMBs on a rotator, by imaging the cells using an upright microscope (BX63, Olympus, Japan) using a 100× oil immersion lens and z-stack imaging. Control groups included NTC, US treatment without TMBs, and untargeted MBs + US insonation. Next, TMB concentration per cell was optimized. The TMB concentration tested were 25, 50, and 100 TMBs per cell. Experiments were carried with a constant treatment duration of 30 s. In addition to the previously mentioned control group, this experiment also included a control group of cells + TMB only (without insonation) for a ratio of 50 and 100 TMBs per cell. Finally, cell viability as a function of the PNP was optimized as a function of US center frequency (80 and 250 kHz). After treatment, cells were transferred to a six-well tissue culture dishes already containing RPMI 1640 complete medium supplemented with 2.5% v/v penicillin–streptomycin. Cells were cultured at 37 °C in a humidified 5% CO_2_ incubator for 72 h and were collected in 500 μL of TrypLE Express. Hemocytometry with Trypan Blue dead cell exclusion was used to assess viable cell number. All treatments were analyzed in triplicate.

### Breast Cancer Animal Model

Female FVB/NHanHsd mice (8 to 12 weeks old, 20–25 g, Envigo, Jerusalem, Israel) were used as the breast cancer animal model. Met-1 mouse breast carcinoma cells were a gift from Prof. Jeffrey Pollard, University of Edinburgh, Edinburgh, UK, and Prof. Neta Erez, Tel Aviv University, Tel Aviv, Israel. Met-1 cell line^65^ was cultured in Dulbecco modified Eagle medium (DMEM, high glucose, supplemented with 10% v/v fetal bovine serum, 1% v/v penicillin–streptomycin and 0.11 g/L sodium pyruvate) at 37 °C in a humidified 5% CO_2_ incubator until about 85% confluency on the day of the injection. Cells were then collected via dissociation with TrypLE Express and resuspended at 1 × 10^6^ cells in 25 μL PBS+/+ for bilateral subcutaneous injection into #4 and #9 inguinal mammary fat pad to obtain primary tumor model. Tumor size was recorded every 4 days until they reached approximatively 4 mm in diameter (approximately 14 days after cell injections). All animal procedures were performed according to guidelines of the Institutional Animal Research Ethical Committee.

### In Vivo Ablation Treatment

A total of 28 bilateral FVB/NHanHsd tumor-bearing mice were studied. The 250/80 kHz spherically focused single-element transducer was placed at the bottom of a degassed water tank facing upward and aligned to focus at an agar spacer which positioned the tumor at the focal depth of the transducer (*z* = 45 mm). The agar spacer was prepared as previously described for the agar cube. Anesthesia was induced with 2% isoflurane in ambient air (180 mL/min), and the treated area was shaved and fur further removed using a depilatory cream for a better coupling. The mouse was positioned on its side, on top of the agar spacer, and US gel was used for coupling. Before the ablation treatment, 2 × 10^7^ TMBs in 20 μL degassed PBS solution were IT injected. The TMBs solution was freshly prepared before each IT injection. For the 250 and 80 kHz center frequency treatments, a PNP of 800 kPa (MI of 1.6) and 250 kPa (MI of 0.9) was applied, respectively. The parameters were chosen such that the CI for both frequencies will remain similar (∼3.2), while the MI remained below the 1.9 guideline. For both frequencies, 125 cycles of a sinusoid US signal with a PRF of 30 Hz and a total duration of 1 min were applied. The TMBs tumor distribution before and after treatment was assessed by US imaging in Vevo 2100 US system. Control groups included NTC, TMBs only (without US treatment), and US only. Bilateral tumor-bearing mice were sacrificed 1 day after US mediated ablation for tumor removal and histology analysis. Frozen tumors were cryo-sectioned to 12-μm-thick slices and stained with hematoxylin (Leica 3801542) and eosin (Leica 3801602) (H&E) according to a standard procedure. The H&E slides were scanned using the Aperio Versa 200 slide scanner (Leica Biosystems, Buffalo Grove, IL) at 20× optical magnification.

### Statistics

Statistical analyses were performed using Prism9 software (GraphPad Software Inc.). Results are presented as mean ± SD. Statistical tests are reported in the relevant captions. *P* values less than 0.05 were considered significant and were adjusted for multiple comparisons as indicated in the captions.

## Supporting Information Available

Normalized contrast reduction as a function of the peak negative pressure; Optimization of the number of ultrasound (US) cycles and the pulse repetition frequency (PRF) in a tissue mimicking phantom (PDF)
