# Supplementary material for: Acoustically Detonated Microbubbles Coupled with Low Frequency Insonation: Multiparameter Evaluation of Low Energy Mechanical Ablation
Source: Bioconjug Chem. 2021 Jul 19;33(6):1069–79. doi: 10.1021/acs.bioconjchem.1c00203 (PMC9204695; doi:10.1021/acs.bioconjchem.1c00203)
Supplement: Supplementary file 1 — bc1c00203_si_001.pdf [file bc1c00203_si_001.pdf]

Supporting information for

Acoustically detonated microbubbles coupled with low frequency insonation: Multiparameter evaluation of low energy mechanical ablation

Mike Bismuth[†], Sharon Katz[†‡], Hagar Rosenblatt[†], Maayan Twito[†], Ramona Aronovich[†], and Tali Ilovitsh[†‡*]

[†] Department of Biomedical Engineering, Tel Aviv University, Tel Aviv 6997801, Israel

[‡] The Sagol School of Neuroscience, Tel Aviv University, Tel Aviv 6997801, Israel

*Corresponding Author
E-mail address: ilovitsh@tauex.tau.ac.il

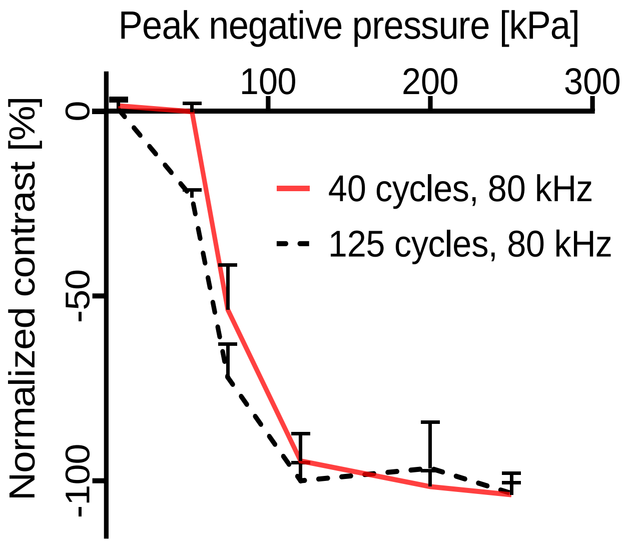

Figure S1. Normalized contrast reduction as a function of the peak negative pressure for 80 kHz insonation with 40 cycles or 125 cycles, and a pulse repetition frequency of 30 Hz. All experiments were performed in triplicates. All data are plotted as mean ± SD.

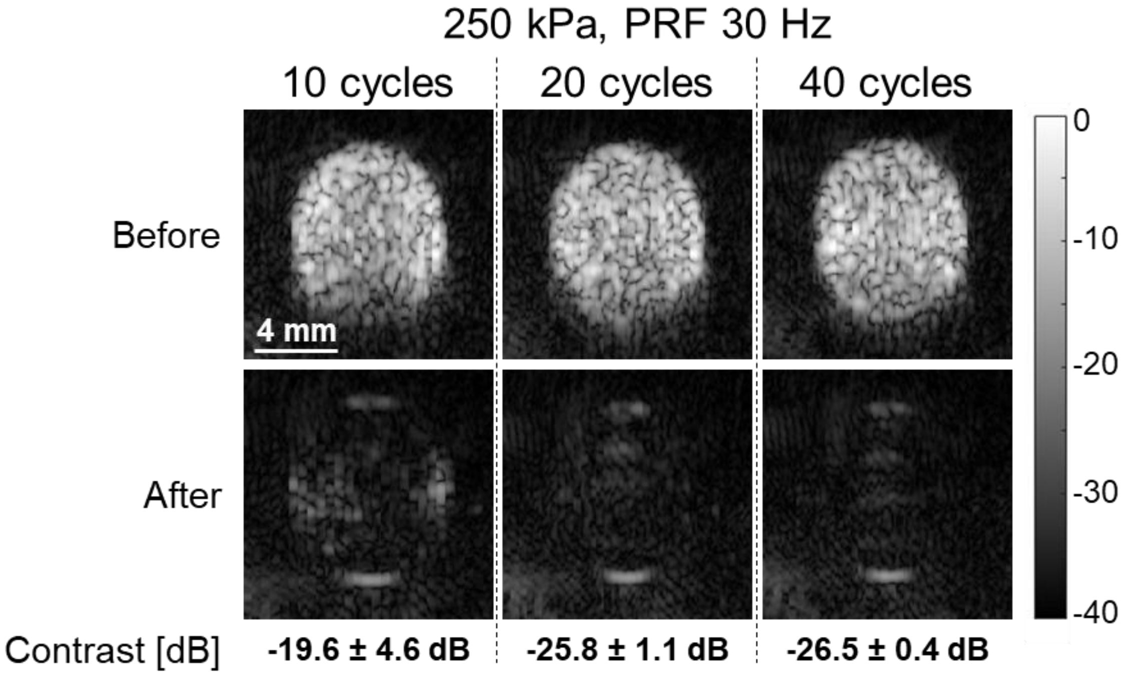

Figure S2. Optimization of the number of ultrasound (US) cycles in a tissue mimicking phantom. US images of a microbubbles-filled inclusion before and after application of a therapeutic US treatment with center frequency of 80 kHz, for number of cycles of 10, 20 and 40 (duty cycles of 0.375%, 0.75% and 1.5%, respectively). Constant parameters are peak negative pressure of 250 kPa and pulse repetition frequency (PRF) of 30 Hz. All experiments were performed in triplicates. All data are reported as mean ± SD.

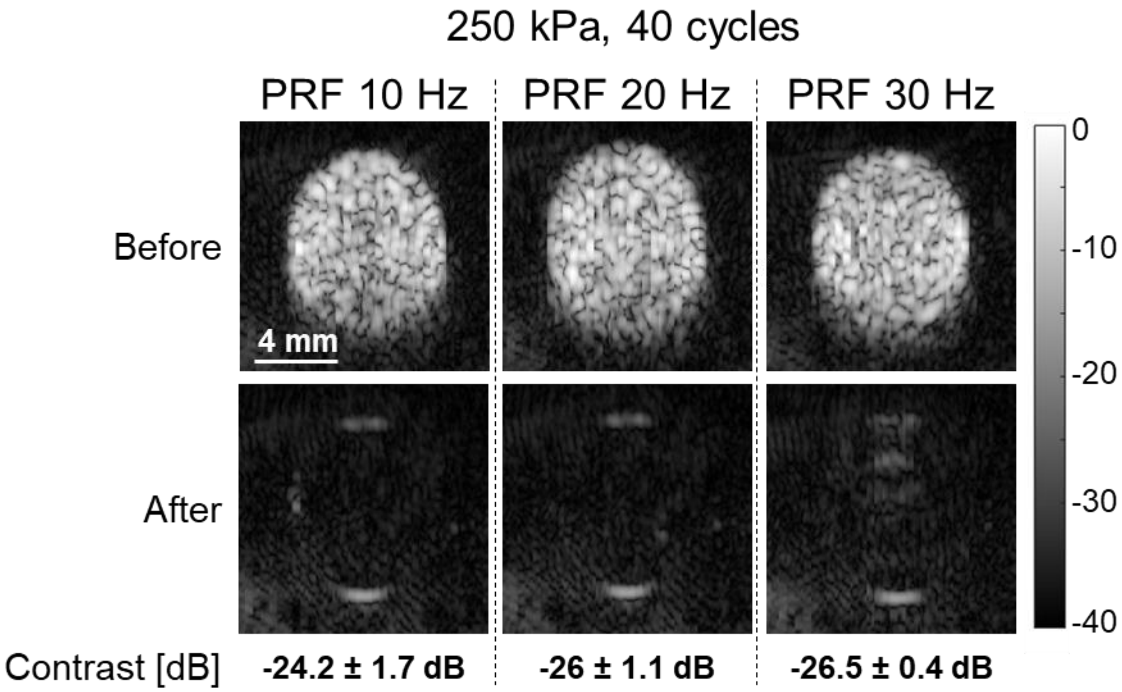

Figure S3. Optimization of the pulse repetition frequency (PRF) in a tissue mimicking phantom. Ultrasound (US) images of a microbubbles-filled inclusion before and after application of a therapeutic US treatment with center frequency of 80 kHz, for PRFs of 10, 20 and 30 (duty cycles of 0.5%, 1% and 1.5%, respectively). Constant parameters are peak negative pressure of 250 kPa and pulse length of 40 cycles. All experiments were performed in triplicates. All data are reported as mean ± SD.
